# The case for balloon eustachian tuboplasty in children

**DOI:** 10.1097/MOO.0000000000000991

**Published:** 2024-07-12

**Authors:** Joonas Toivonen, Dennis Poe

**Affiliations:** a Department of Otorhinolaryngology – Head and Neck Surgery, Turku University Hospital and University of Turku, Turku, Finland; b Department of Otolaryngology and Communication Enhancement, Boston Children's Hospital and Harvard Medical School, Boston, Massachusetts, USA

**Keywords:** balloon dilation, eustachian tube, obstructive dysfunction, patulous dysfunction, pediatric

## Abstract

**Purpose of review:**

Balloon dilation of the cartilaginous portion of the Eustachian tube has increasingly gained acceptance among otolaryngologists in the treatment of obstructive Eustachian tube dysfunction. There is however little data on the procedure performed in children. The purpose of this study is to review the recent developments regarding balloon dilation in pediatric patients.

**Recent findings:**

Balloon dilation of the Eustachian tube is safe in pediatric patients. The effects of the procedure are durable during long term follow-up. Diagnosing obstructive dysfunction remains challenging. There is no single test or questionnaire for diagnosing the condition; instead a series of appropriate tests should be used. The pediatric Eustachian tube is very responsive to the effects of balloon dilation. While the treatment is effective, overtreatment can have unwanted results such as patulous symptoms. Reducing the time of dilation should therefore be considered.

**Summary:**

Otolaryngologists performing the procedure should be familiar with the effects of balloon dilation on the pediatric Eustachian tube and consider altering the duration of dilation accordingly. Further studies are needed especially regarding patient selection, optimal age for dilation and balloon parameters for pediatrics (e.g. dimensions, inflation duration, inflation pressure).

## INTRODUCTION

**FIGURE 1:**
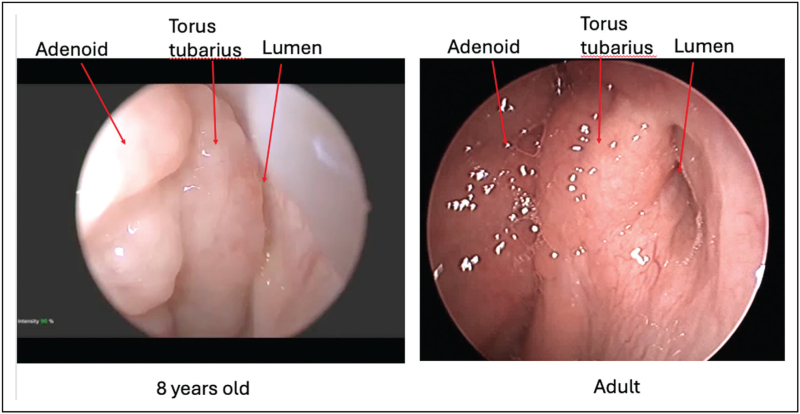
Comparison of pediatric and adult left Eustachian tubes.

Obstructive Eustachian tube dysfunction (OETD) has been reported to have an estimated prevalence ranging from 4.4% to 6.1% in adolescents in the United States [1,2]. Balloon dilation of the Eustachian tube (BDET) has become accepted in the management of refractory OETD in adults [3,4]. In children, OETD has traditionally been treated indirectly with adenoidectomy and tympanostomy tubes (TT). Regarding BDET for pediatric patients, there is discussion over indications and patient selection including minimum age for dilation, duration of dilation, and potential risks. The clinical consensus statement from the American Academy of Otolaryngology – Head and Neck Surgery provides a comprehensive guideline on the criteria for BDET [5]. They agreed on the necessity of history, otoscopy, tympanometry, pure tone audiometry, and endoscopic investigation of the nasopharynx prior to considering BDET. With pediatric patients, endoscopic evaluation of the nasopharynx and the ET orifice (whether in the office or intraoperatively) is especially important since there is a high proportion of adenoid hypertrophy with associated hypertrophy of the torus tubarius affecting Eustachian tube (ET) function [6] (Fig. 1).

The diagnosis of Eustachian tube dysfunction should always be based on both patient reported symptoms and objective findings [7]. For determining the diagnosis, neither subjective symptoms nor any current single test alone is enough. Shortcomings of the current ET function tests either with reliability, practicality, or correlation with clinical presentation has hindered the acceptance although several tests have been developed [8].

For adults and children alike, surgical treatment may be considered if appropriate medical management has failed to adequately treat persistent ETD. Conditions including chronic rhinosinusitis, turbinate hypertrophy, nasal septal deviation or adenoid hypertrophy possibly contributing to OETD, can be considered for operative treatment if indicated [9]. If endoscopic evaluation suggests that the primary difficulty in the opening of the ET valve is due to adenoid or tubal tonsil hypertrophy, adenoidectomy and trimming of the tubal tonsil tissue may be the only intervention needed [10]. 

**Box 1:**
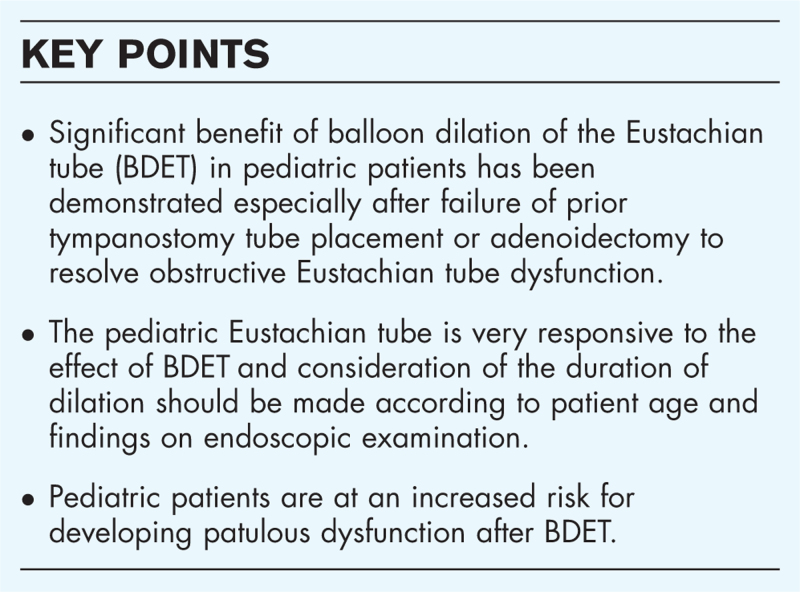
no caption available

### Patient selection/diagnostics

With pediatric patients, an ongoing otologic condition such as negative middle ear pressure or effusion with a need for repeat tympanostomy tube (TT) insertions, tympanic membrane (TM) perforation, TM retraction or retraction pocket, or a failed tympanoplasty often suggests a failure in ET function. Young patients may not necessarily report symptoms specific to a diagnosis. When conventional therapies have not resulted in resolution of symptoms or signs, operative treatments can be considered. Developmental anatomy of the pediatric ET including ET length, length of the cartilaginous ET, and ET horizontal angle was studied by Magro *et al.* from CT scans. They found the ET to mostly reach adult size by the age of eight although some parameters mature even earlier [11].

Alper *et al.* studied the use of various ET function tests in children including the forced response test (FRT), inflation-deflation test, pressure chamber test, Valsalva, Toynbee, sniff, and dive maneuvers in addition to an otolaryngologic examination including otomicroscopy, pneumatic oto-videoendoscopy, and nasopharyngeal videoendoscopic evaluation of the ET orifice during swallow and other maneuvers. They also employed special devices such as the tubomanometer, which is not available in most centers. Their use in USA is restricted to research purposes. Also, the diagnostic accuracy of tubomanometry has not been established, especially in the pediatric population. They concluded that until the availability of more sensitive and specific questionnaires and tests with high diagnostic accuracy are available for widespread clinical use, awareness to the wide presentation of ETD and the best assessment methods available to each physician should be used [12]. Any questionnaires on patient symptoms are expected to have limited reliability in children, especially when parents are the responders [12]. Existing validated questionnaires regarding ET function are all patient reported outcomes measures (PROM) instruments and are not intended for diagnosis as they do not distinguish between other differential diagnoses of aural fullness.

Current reports on BDET in children have used variable indications including chronic otitis media with effusion, chronic TM perforation, adhesive otitis media, recurrent otitis media, and barochallenge induced ETD, all refractory to medical management and usually after TT placement and adenoidectomy [13,14].

The effect of an enlarged adenoid on ET function has been recognized in early studies especially in children with recurrent or persistent otitis media with effusion (OME) or ETD [15].

### Special considerations

BDET may be performed with an endoscope passed transnasally through the ipsilateral or contralateral nasal cavity and the balloon passed ipsilaterally. In smaller children, contralateral placement of the endoscope is more commonly employed.

**FIGURE 2:**
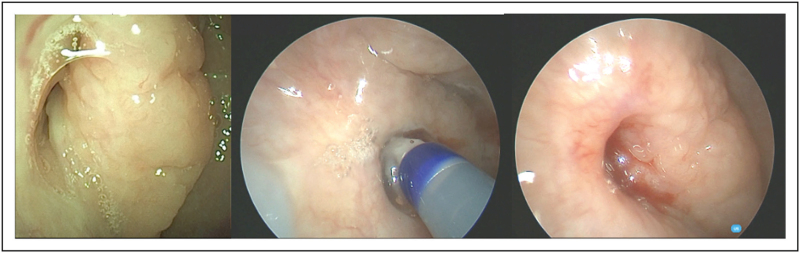
Balloon dilation of right Eustachian tube, 10 year old. (a) Preop. (b) Balloon inflated. (c) Immediately postinflation.

For some of the younger children, due to the angle and length of the guide catheter, nasal anatomy may not allow for easy direction of the balloon catheter into the tubal orifice transnasally. In that instance, a transoral approach can be used passing the balloon catheter through a suitable introducer, such as an olive tipped suction, up to the lumen of the ET with the endoscope in the nasal cavity for viewing [16,17^▪▪^] (Fig. 2).

Yu *et al.* studied the lengths of the cartilaginous ET through development by CT scan and they offered the possibility to estimate ET length in pediatric patients in case there is no imaging available. Average lengths were 21 mm (range 13.5–25.8) under age 2, 24 mm (range 18.4–30.0) at ages 2–5, 25 mm (range 21.3–31.7) at ages 6–10, and 27 mm (range 21.8–33.5) at age ≥ 15. They found that mean lengths across all ages 0–19 years showed that females had a significantly shorter cartilaginous ET at 24.1 mm compared to males at 25.8 mm. If an insertion depth of 20 mm were to be used as an estimated limiting insertion depth for all patients, children aged 5 and under could be at risk for insertion of a balloon into the bony ET given their potential ET length of under 20 mm. They also provided a model for calculating ET length if no imaging is available based on either patient height, weight, age or body-mass index (BMI). Prediction of ET length based on patient height was reliable and easiest to calculate [18].

A systematic review of adverse events from BDET reported 98 complications in 7155 patients (1.4% incidence) and most were self-limited. The most commonly reported events were subcutaneous emphysema and pneumomediastinum followed by epistaxis and acute otitis media [19^▪^].

The most serious potential complication from BDET was injury to the internal carotid artery which is located in proximity to the cartilaginous and bony ET [20]. A case of carotid dissection presenting with a stroke seven days after BDET was reported involving a catheter using a metal rail-guided balloon [21]. The patient made a full recovery after receiving a stent. While the case was an adult patient, there is a potential risk for the complication in pediatric patients also.

False passage through the thin membranous (anterolateral) wall of the cartilaginous ET is the most likely cause of subcutaneous emphysema, pneumomediastinum and the reported carotid injury. In the authors’ experience with teaching courses using cadavers, false passages have occurred when participants inserted the catheters without seeing and following the course of the membranous wall, which curves medially before turning laterally toward the ear. Aligning a catheter with the torus tubarius, which is more easily seen with a zero degree view-angled endoscope, may result in penetration of the very thin membranous wall. In the instances of false passage that were recognized in training lab sessions, surgeons have never had tactile feedback when it occurred. Once a catheter has created a false passage, it introduces the risks of subcutaneous emphysema, pneumomediastinum and failure of the balloon inflation to the properly expand within the lumen of the ET. Furthermore, the course of a false passage leads directly toward the extratemporal internal carotid artery, placing it at risk for blunt or penetrating trauma.

Treble and colleagues studied the risk of entering the middle ear during BDET with a 3.3 × 20 mm balloon catheter in an adult cadaver model [22]. They found that using the manufacturer's recommended technique, the catheters entered the middle ear in all 16 ETs showing that certain devices with an outside diameter small enough to pass through the bony-cartilaginous isthmus, may enter and potentially damage middle ear and inner ear structures. Measuring a planned insertion depth on the catheter and inserting visibly to that depth was recommended. Sensorineural hearing loss and tinnitus have been reported with the 3.3 mm diameter balloon [23]. The cadaver models were of adult size and the potentially shorter length of the pediatric ET should be considered regarding the risk.

Howard *et al.* analyzed the safety of BDET in a pediatric cohort of 42 patients. In their study, two children had a minor adverse event with a complication rate of 4.7% (epistaxis and vestibular migraine [14]. Another study reported two cases of hemotympanum after BDET that resolved without intervention within a week out of 55 dilated ETs [24]. Development of patulous Eustachian tube dysfunction (PETD) is a potential risk with BDET. In a study by Hubbell *et al.* the incidence of patulous symptoms after balloon dilation was 7% using a 6 mm diameter balloon device. Most cases were self-limited, but 4/20 patients with patulous symptoms had persistent symptoms, one of whom had successful surgery with mass loading of the tympanic membrane. They found that pediatric patients (age 7–18) were at greater risk of developing patulous symptoms (13.3%) than adult patients (5.6%, age 19–49 years and 2.1%, age ≥50); *P* = 0.01 [17^▪▪^].

### Outcomes

Children are commonly affected by chronic OME and given the growth and maturation potential of the ET with time, tympanostomy tube placement is still considered the first line treatment. Balloon dilation has mainly been studied as a treatment modality for recalcitrant disease, but some studies have also reported results using it as an alternative first line treatment.

Tisch *et al.* in 2013 were the first to report the use of BDET in pediatric patients [25]. A subsequent report of 299 BDETs in 167 children aged 4–12 years by Tisch *et al.* showed significant improvement in tympanogram results with 39% of patients having a type A tympanogram postoperatively compared to 11% preoperatively (*P* < 0.001), TM appearance with 66% with a normal TM finding postoperatively compared to 17% preoperatively, and hearing thresholds with the median air conduction threshold at 1 kHz improving from 20 dB to 10 dB after BDET (*P* < 0.001). All patients had previously has a procedure done to improve their OETD with TT placement and adenoidectomy being the most common. They also collected data on the satisfaction of the family on treatment results with 80.1% being satisfied with treatment results [26].

In a meta-analysis of 408 pediatric BDET patients, Aboueisha *et al.* found BDET with or without TT placement to produce significantly improved outcomes (including decrease in type B tympanograms from 64.2% preoperatively to 16.1% postoperatively) compared to TT placement alone. They concluded BDET to be comparable, if not superior, to ventilation tube insertion when treating chronic otitis media with effusion [27].

In another study, BDET performed with or without other procedures as indicated, significant improvement in otomicroscopic findings, tympanograms, mucosal inflammation scores, audiometric results and the ability to perform a Valsalva maneuver was observed. At 12 months, TMs were healthy on otomicroscopy in 55% of patients compared to 9% preoperatively (*P* < 0.001). Tympanogram was type A at 12 months in 59% of patients compared to 23% preoperatively (*P* < 0.001). Mucosal inflammation scores improved from a preoperative mean of 3.2–1.2 at 24 months (*P* < 0.001). Patients undergoing BDET were also compared with matched, controlled patients undergoing repeat TT placement, most of whom had also undergone adenoidectomy. Patients undergoing TT placement were more likely to need further TT placement in the treatment of their OETD with a 56% probability of being failure free at two years compared to the BDET group at 87% [16].

In a previous study by Leichtle *et al.*, BDET was performed on 52 children (97 ears) refractory to conventional treatment, and the authors found improvement in type A tympanograms from 14% preoperatively to 50% at 1 year postoperatively. Patients also reported improvement in the ability to perform the Valsalva maneuver from 13% preoperatively to 60% postoperatively [28].

A study comparing BDET together with tympanostomy tube placement and tympanostomy tube placement alone in children was done by Chen *et al.*[29]. They found that ETD symptoms resolved in 94% of the children in the BDET group and 89% in the control group with no significant difference between groups. Gurberg *et al.* recently reported on long term results of BDET with a mean follow up of 6.7 years. Patients aged 14 months to 14 years underwent BDET and were compared with patients matched by age, sex, number of prior TTs, and prior adenoidectomy who received TT placement. The probability of being failure free at six years was 88% in the BDET group and 53% in the TT group [30].

Demir and Batman compared the effect of either BDET or tympanostomy tube insertion as first line treatment on the quality of life in children with a mean age of 7 years [24]. Both groups improved significantly at the six week and one year follow-ups, but not to the advantage of either group. They also measured the quality of life with the OM-6 questionnaire. At the 6 week follow up the improvement in the TT group was significantly higher suggesting a more rapid improvement in symptoms but there was no difference at the 12 month follow up. According to the results, BDET could be considered as an alternative as first line treatment but not superior to tympanostomy tubes. However, in patients being considered for repeat TT placement having had prior TT and adenoidectomy, there may be an advantage to performing BDET.

### Other pediatric considerations

Young patients aged 18 years or under have been shown to be at an increased risk of developing PETD. Severe ET mucosal inflammation score was also found to be a risk factor for postoperative PETD. These may be due to a greater sensitivity of the inflamed mucosa to the effects of dilation, given that inflammatory mediators in the upper respiratory tract are typically upregulated more in pediatric patients than in adults. ET anatomy and ET size relative to the balloon may also contribute to the risk of PETD, but the increased risk was also noticed in patients aged 12 to 18 when the ET should have reached adult size. Excessive duration of dilation could be a potential risk factor for developing PETD in patients with greater susceptibility for patulous symptoms. The senior author has found anecdotally that the incidence of developing PETD with balloon dilation has markedly reduced since limiting duration time to 1.5 min or less for pediatric patients. Duration of dilation is otherwise recommended to be commensurate with the severity of inflammatory disease seen on preoperative endoscopy [17^▪▪^].

Decreasing the maximum dilation pressure could be an alternative adjustment, but no data on its effectiveness is currently available. Smaller balloon size for younger patients could be considered depending on regional availability and approval for use.

It is a common phenomenon that patients with longstanding OETD may develop PETD due to atrophy of overstimulated mucosa and submucosa within the functional valve of the lumen of the ET. Chronic allergic rhinitis has been shown to be the most common co-morbidity associated with PETD and development of PETD after OETD must be considered prior to offering BDET. Patients who develop PETD begin to sniff frequently in an attempt to minimize patulous symptoms and they may generate negative pressures sufficient to cause retraction of the TM, middle ear effusion, fixed retraction pockets and even cholesteatoma [31]. These patients may be treated with tympanostomy tubes for a diagnosis of OETD, but the tube may be effective in relieving their patulous symptoms. When patulous symptoms recur after extrusion of the tube, additional tubes may be offered and these patients may have a history of repeated TT placement. Before considering performance of BDET in patients, it is necessary to ask about sniffing to control autophony of voice or breathing. BDET on patients with PETD can exacerbate their condition.

## CONCLUSION

Careful selection of patients for BDET is necessary for optimal outcomes. Systematic reviews and matched controlled studies have demonstrated significant benefit of BDET in pediatric patients, especially after failure of prior TT or adenoidectomy to resolve OETD. Further studies are needed to refine the expectations for outcomes, with and without adjunctive procedures, and to investigate what should be the criteria or limitations for use in children under age 8. The potential of BDET as a first line treatment requires further studying although it is appropriate in patients with barochallenge in whom TT placement is not desired. Further studies on parameters of dilation time, inflation pressure and balloon size for pediatric patients is needed in order to minimize potential complications while achieving optimally efficacious and durable results.

## Acknowledgements

*None.*

### Financial support and sponsorship

*This work received no specific funding.*

### Conflicts of interest

*Dennis Poe is a consultant for Acclarent.*

## REFERENCES AND RECOMMENDED READING

Papers of particular interest, published within the annual period of review, have been highlighted as:

▪ of special interest
▪▪ of outstanding interest
